# Pelvic actinomycosis presenting with a large abscess and bowel stenosis with marked response to conservative treatment: a case report

**DOI:** 10.1186/1752-1947-1-141

**Published:** 2007-11-21

**Authors:** Hiroaki Nozawa, Yoshinao Yamada, Yasuhiko Muto, Shirane Arita, Kohzo Aisaka

**Affiliations:** 1 Department of Surgery, Odaira Memorial Tokyo Hitachi Hospital, Tokyo, Japan; 2 Department of Gynecology, Odaira Memorial Tokyo Hitachi Hospital, Tokyo, Japan

## Abstract

Pelvic actinomycosis is a rare disease that can result in abscess formation, bowel obstruction, and other serious complications. Moreover, the correct diagnosis can seldom be established before radical surgery because the disease often mimics pelvic neoplasms. It has been recently recognized that pelvic actinomycosis is associated with long-term use of an intrauterine contraceptive device.

We report a woman with a long-standing intrauterine contraceptive device who visited our hospital complaining of symptoms mimicking large bowel ileus with a subacute course. X-ray fluorography and sigmoidoscopy showed marked stenosis in the sigmoid colon but rejected the possibility of colon cancers. Abdomino-pelvic CT and MRI revealed a huge abscess lying over the urinary bladder and anterior to the uterus. Furthermore, a cervical Papanicolaou smear disclosed *Actinomyces *species. We removed the intrauterine device from the patient. Subsequent high-dose ampicillin administration led to dramatic shrinkage of the abscess and improved the management of the bowel movement quickly. This is a successful case of symptomatic pelvic actinomycosis that was correctly diagnosed and treated without unnecessary surgical intervention.

## Case presentation

A 51-year-old woman, gravida 2, para 2, visited a local clinic and presented with constipation, worsening abdominal cramps, nausea and increased abdominal girth for a month. She was initially diagnosed with enterocolitis, but became progressively more constipated. Two weeks later, she was referred to our hospital for further examinations, having received fosfomycin for the previous 4 days aimed at alleviating her elevated white blood cell (WBC) count (12,700/μl) with a raised C-reactive protein (CRP) level of 11.3 mg/dl.

The patient was admitted to the hospital to assess the cause of bowel obstructive symptoms and to treat malnutrition. Her body weight had decreased by 7 kg over a month compared to when in a healthy condition (40 kg). Physical examination on admission revealed tenderness in the lower abdomen with tympanic bowel sounds but no palpable mass. Her body temperature was 37.0°C. Laboratory tests revealed leucocytosis of 12,000/μl with a left shift (Seg 87%), mild anemia (hemoglobin: 11.9 mg/dl), CRP level of 6.99 mg/dl and erythrocyte sedimentation rate (ESR) of 70.0 mm/h. Serum tumor markers were all normal: carcinoembryonic antigen was 1.4 ng/ml, CA19-9 12.6 U/ml, and CA-125 28.4 U/ml. Moreover, no abnormal findings were seen in liver and renal function tests.

**Figure 1:**
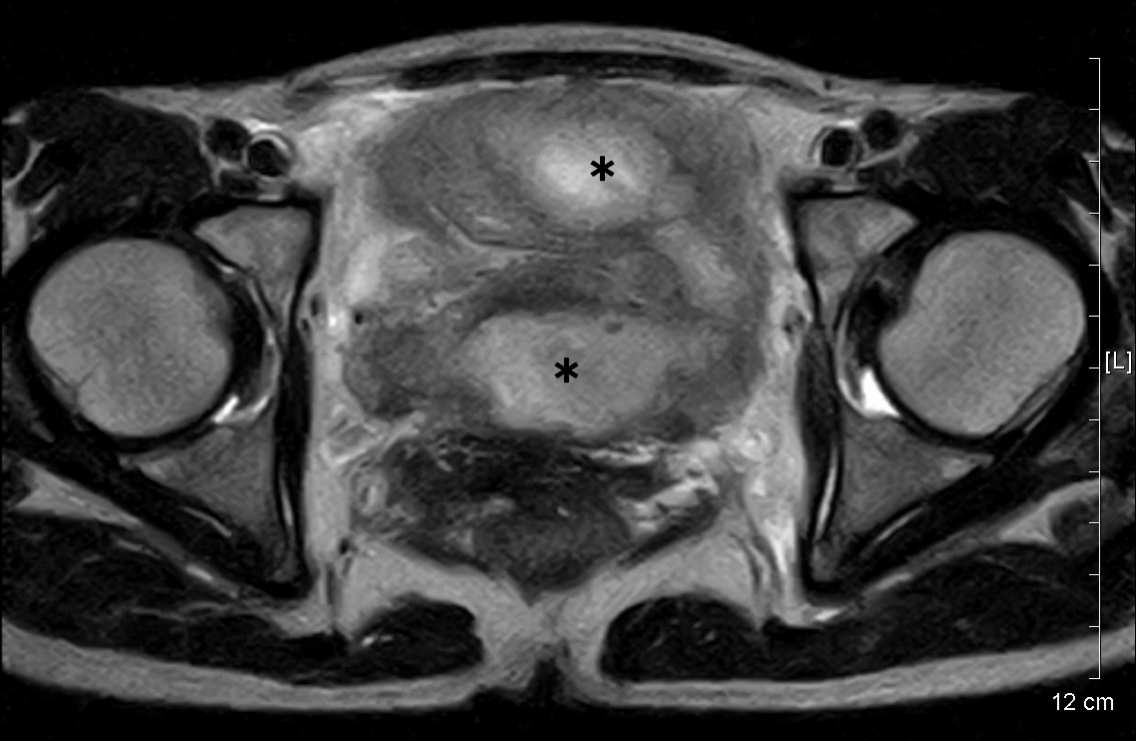
An oblique magnetic resonance T2-weighted image disclosed a horseshoe-shaped abscess, 8 × 7 cm in size (indicated by asterisks). The abscess wall is of intermediate to slightly high intensity whereas the fluid inside exhibits high intensity signal.

**Figure 2:**
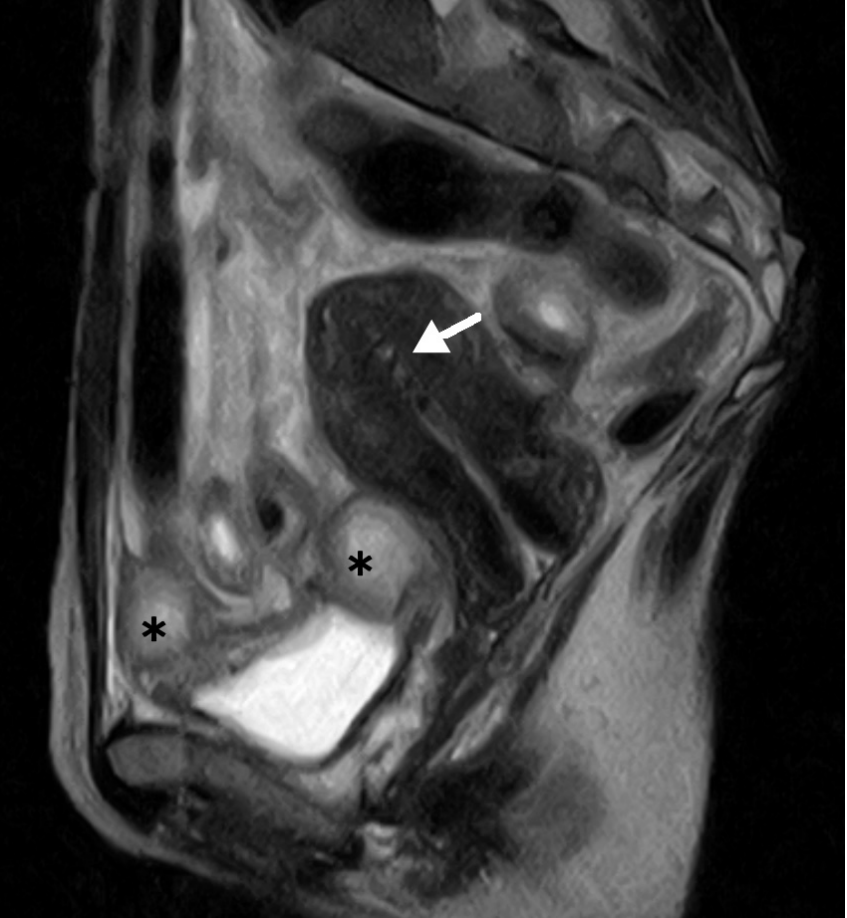
A sagittal section visualized a foreign body that was later identified as an IUD (indicated by a white arrow) inside the uterus with non-homogeneous signal levels. The peritoneal abscess (indicated by asterisks) is just above the urinary bladder. The bladder wall is also thickened.

Contrast X-ray fluorography by enema showed a crooked and narrow segment of the sigmoid colon; however, the lumen looked relatively smooth at the stenotic site. Flexible sigmoidoscopy revealed luminal narrowing at a curved site around 25 cm from the anus without obvious mucosal abnormality. Loss of flexibility of the sigmoid colon suggested possible pelvic inflammation. Abdomino-pelvic computed tomography (CT) images showed raised fat density in the mesentery in front of the uterus, consistent with our impression. Moreover, a vast space of fluid density above the urinary bladder suggested an abscess. Magnetic resonance imaging (MRI) also demonstrated an abscess, 7 – 8 cm in size, of intermediate to slightly high intensity signal on T2-weighted images (fig 1). The ceiling wall of the urinary bladder was also thick, suggesting extensive pelvic inflammation. Furthermore, a foreign body, presumed to be an IUD, was visualized inside the uterus with heterogeneous signal levels (fig 2).

**Figure 3:**
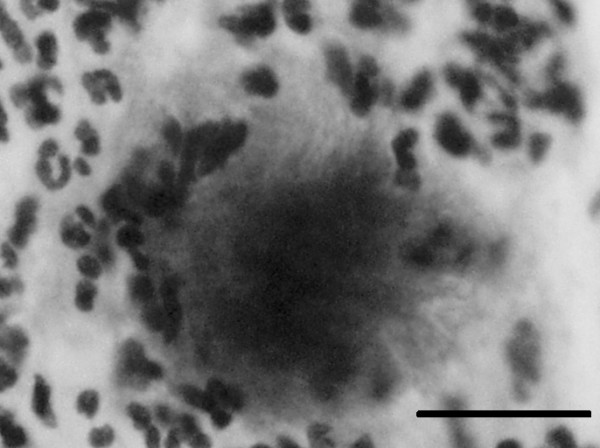
A typical bacterial aggregate from a cervical Papanicolaou smear. It comprises a cotton ball-like colony with protruding mycelial filaments, suggesting *Actinomyces *species infection. The bar indicates 20 μm.

Further gynecological inquiries unveiled that the patient had been using IUDs for 21 years; she had changed IUDs twice in the past but the most recent had been *in situ *for 15 years. She had experienced prolonged menstruation (about 21 days) and increased vaginal discharge recently. A cervical Papanicolaou smear showed dark cotton ball-like bacterial colonies with protruding a filamentous structure, highly suggestive of *Actinomyces *species infection (fig 3).

Based on the radiological images and cytopathological result, we envisaged that her symptoms were primarily caused by pelvic *Actinomyces *infection associated with the long-standing IUD. Eight days after admission, the IUD was removed, while intravenous administration of ampicillin (4 g/day) was initiated.

Her recovery was quite good; WBC count and CRP returned to normal levels in a week by antibiotic treatment. With the help of a mild laxative, her abdominal distension and tenderness gradually ameliorated. Soon she was capable of eating as much as previously and was discharged on hospital day 18. The patient was switched to oral ampicillin for a month. Repeated MRI showed that the pelvic abscess had mostly disappeared.

## Discussion

*Actinomyces *is a slow-growing, filamentous, gram-positive anaerobe. Traditionally actinomycosis of the female genital tract has been thought to originate from ascending infection of the bacteria by oro- or anogenital contact or by a pessary. The association between IUDs and pelvic actinomycosis was first described around 1970 [1,2], and has now been well documented [3-5]. Overgrowth of *Actinomyces *together with alteration of the genital tract environment and/or breached uterine mucosal barrier by mechanical means may develop endometritis and pelvic actinomycosis.

Fiorino reviewed 92 cases of pelvic actinomycosis. The patients were usually in their reproductive years with a history of having an IUD for 8 years on average. Multiple symptoms can appear in varied frequencies, such as abdominal pain (85%), body weight loss (44%), fever (60%), and vaginal discharge (24%). On laboratory tests, 70% of the patients were anemic, 76% had leucocytosis, and 95% had accelerated ESR [3]. Because most of the aforementioned abnormalities were observed on admission, our patient was a typical case of pelvic actinomycosis. Notably, IUD implantation had continued for 21 years, a very long duration that is thought to have a higher risk of developing pelvic actinomycosis [2].

The usual CT findings of pelvic actinomycosis include abscess, an infiltrative mass with dense and non-homogenous contrast enhancement, and bowel wall thickness [5-7]. By contrast, there have been only a few reports on MRI findings of pelvic actinomycosis [6,8], describing that *Actinomyces*-associated masses were of intermediate signal intensity on T1-weighted images and of intermediate to low signal intensity on T2-weighted sequences, whereas cystic components exhibited a high T2 signal [6,8]. Unlike mass-forming actinomycosis, we observed that the wall of abscess-forming actinomycosis was of intermediate to high signal intensity on T2-weighted MR images.

Unfortunately, these image findings of actinomycosis appear non-specific [5] and are dependent on the involved regions and degree of inflammation [7]. Together with the clinical symptoms, the images often simulate those of gastrointestinal and gynecological malignancies [3,4]. These reasons render the disease undiagnosable, and 83% of cases undergo major surgical operation such as hysterectomy, salpingo-oophoerectomy, bowel resection, drainage of abscess, etc [3,7]. Hence, the most crucial stage of this uncommon disease is to make a correct diagnosis. In this regard, Gupta *et al. *underscored the importance of cervical Papanicolaou smears, immunofluorescent staining and anaerobic culture in the diagnosis of actinomycosis [9]. CT- or ultrasound-guided aspiration or needle biopsy may be useful to reach a diagnosis [4,5]. In our case, causative colorectal neoplasm(s) were unlikely from the results of X-ray fluorography and sigmoidoscopy, and we rather suspected infection. The image findings in our patient were essentially in accordance with the features of pelvic actinomycosis but were not distinct from other inflammatory diseases. The most helpful evidence to confirm the diagnosis was typical aggregates of *Actinomyces *species detected in a cervical smear [4].

Once diagnosed, pelvic actinomycosis should be treated primarily by penicillin administration as well as IUD removal [7]. Tetracycline, clindamycin and erythromycin can alternatively be used for patients allergic to penicillin [3]. Even when a pelvic abscess is present, conservative medical treatment is considered the first-line therapy unless it is large, for instance, more than 8 cm in diameter, which often requires surgical drainage [4]. Although our patient manifested severe gastrointestinal symptoms and an abscess close to 8 cm in diameter, we explored the opportunity to avoid unnecessary drainage. She responded to the conservative therapy rapidly and remarkably, which was confirmed by follow-up laboratory tests and MRI.

## Conclusion

Currently, IUDs are one of the most common methods of reversible contraception worldwide [10]. Although IUDs are highly effective for preventing unintended pregnancy, users and medical doctors should remember the risk of complications, including pelvic inflammatory disease [10]. Deliberate examinations, careful evaluation of results and images, and a cooperative and appropriate treatment strategy spared our patient from radical interventions. Whenever examining IUD users with gastrointestinal symptoms, pelvic actinomycosis should be included in the differential diagnosis [5].

## Abbreviations

CA: cancer antigen;

CRP: C-reactive protein;

CT: computed tomography;

ESR: erythrocyte sedimentation rate;

IUD: intrauterine contraceptive device; 

MRI: magnetic resonance imaging;

WBC: white blood cell.

## Competing interests

The author(s) declare that they have no competing interests.

## Authors' contributions

All authors were involved in the treatment of the patient, and have read and approved the manuscript.

## Consent

Written consent was obtained from the patient for the publication of the case report.
